# Impact of Blanching, Freezing, and Fermentation on Physicochemical, Microbial, and Sensory Quality of Sugar Kelp (*Saccharina latissima*)

**DOI:** 10.3390/foods10102258

**Published:** 2021-09-23

**Authors:** Samuel Akomea-Frempong, Denise I. Skonberg, Mary E. Camire, Jennifer J. Perry

**Affiliations:** School of Food and Agriculture, University of Maine, 5735 Hitchner Hall, Orono, ME 04469, USA; samuel.akomeafrempong@maine.edu (S.A.-F.); denise.skonberg@maine.edu (D.I.S.); camire@maine.edu (M.E.C.)

**Keywords:** seaweed, kelp salad, kelp sauerkraut, acceptability, blanching, microbiology, antioxidant activity, vegetable fermentation

## Abstract

Low seaweed consumption in the West is due to lack of availability and consumer familiarity. In this study, the effects of preservation processes on quality aspects of *Saccharina latissima* products were assessed. First, a blanching (100 °C for 1 or 3 min) treatment was used to produce seaweed salad. In a second study, effects of blanching, freezing, and fermentation on kelp quality were assessed and processed kelp was used to produce sauerkraut. Blanching significantly decreased (*p* ≤ 0.05) the instrumental kelp a* value and firmness. The a* value negatively correlated with overall liking of salads. To prepare sauerkraut, raw, raw/frozen (−20 °C), blanched (100 °C, 1 min), or blanched/frozen kelp were mixed with cabbage, salted, inoculated with starter cultures and fermented. Inconsistent trends in L* values, firmness, and fungi enumeration were observed after fermentation. Consumers evaluated kelp salad (n = 100) and sauerkraut (n = 80) for acceptability. Blanched kelp salad had higher hedonic scores than raw kelp salad. A 100% cabbage sauerkraut control and blanched kelp/cabbage blends were compared; kelp blends were similar to control for appearance, color, and texture but were lower for overall acceptability. Results suggest improved quality and enhanced consumer acceptability of seaweed products with use of minimal processing.

## 1. Introduction

Seaweed cultivation offers potential solutions to environmental challenges, such as eutrophication, by improving water quality [1]. Seaweeds have a higher production rate than terrestrial plants, and they do not require land or fresh water [2]. The sustainability of seaweed cultivation has increased the appeal for their production through aquaculture globally. Moreover, consumers perceive edible seaweed food products as natural and healthy [3]. Seaweeds are rich in dietary fiber, minerals, vitamins, antioxidants, and umami flavor; they can be used in low-calorie diets and serve as functional foods [4,5].

There are numerous seaweed-based products in Asian countries such as China, South Korea, and Japan, with niches of products marketed in Europe and North America. The FAO reported that 290,000 wet tons of seaweed were produced in 2019 in the Americas and Europe [6]. The principal cultivated variety (66%) was kelp, a grouping which encompasses multiple species of brown algae [7,8]. In the U.S., seaweed cultivation is found on the west and east coasts, with Maine and Alaska leading U.S. production (~85%) of about 600,000 wet lbs. of edible seaweed due to their extensive coastlines, as reported by the Island Institute in 2020 [8,9]. The increasing production provides abundant opportunities for industrial development for seaweed consumption. However, little attention has been paid to consumers’ perceptions of seaweed as a food product in the West [10]. Additionally, the extreme seasonality and high perishability of the crop [11,12] may impede the availability of raw materials to produce consumer products without the use of preservation processes.

Prior studies have applied various processes, including drying, freezing, salting, and high-pressure processing, to various seaweed species to increase seaweed product availability throughout the year [11,13,14]. Most of these processes reduced some bioactive compounds and changed the texture of seaweed [15,16]. Blanching prior to some of these preservation methods, including drying and freezing, has been suggested to retard product deterioration rates [17]. Moreover, blanching reduces microbial counts in some vegetables [18] and turns brown seaweed to a bright green color [19]. Processing methods such as fermentation and salting may also add value to seaweed products in addition to providing shelf-life extension.

Fermentation is a low-cost preservation method utilized by some food processors, which increases some bioactive compounds in foods such as cabbage [20], and gives food products unique flavor [21]. Seaweeds can be fermented into a seaweed sauerkraut-style products to create a non-dairy alternative probiotic product for consumers [22,23]. Sugar kelp (*Saccharina latissima*) and winged kelp (*Alaria esculenta*) mixed with cabbage in various ratios were fermented with *Lactobacillus plantarum* (10^6^ CFU/g) and *Leuconostoc mesenteroides* (10^1^ CFU/g) starter cultures to produce seaweed sauerkraut with high lactic acid bacteria levels, which increased as fermentation progressed [23]. Fermentation of sugar kelp with *L. plantarum* for 48 h reduced mercury and cadmium content significantly (*p* < 0.05), as compared to raw kelp [24], which could relieve concerns about heavy metals for health-conscious consumers.

To develop appropriate food products for Western markets from the harvest of domestic seaweeds and also consider seaweed as a vegetable, it is crucial to consider cost-effective preservation methods such as blanching, freezing, and fermentation, which can extend the shelf-life of the raw materials. In the literature available to date, studies on assessment and consumer acceptance of minimally processed seaweed food products are limited. Recent work conducted in our laboratory showed that blanching of sugar kelp resulted in significant changes immediately after treatment, including differences in physicochemical properties of kelp (compared to unblanched samples), particularly color and texture, after 12 months of frozen storage (unpublished data). These significant changes in some of the kelp qualities in response to blanching and/or frozen storage may have a measurable effect on consumer acceptance and may influence commercialization of blanched and/or frozen seaweed food products. Therefore, the hypothesis of this paper was that blanching, freezing, and fermentation may increase kelp quality and consumer acceptability. The effect of these preservation processes on sugar kelp were assessed using physicochemical, sensory, and microbiological methods. To achieve this, two objectives were considered. The first objective of this study was to analyze the effect of blanching (100 °C for 1 or 3 min) on the physicochemical and microbial properties of sugar kelp and to conduct sensory evaluation of a food product (seaweed salad) developed from the blanched kelp, as compared to raw. This was done to determine the effect of minimal processing (blanching) on kelp quality and its impact on consumers’ acceptance. The second study focused on the effects of blanching and freezing on fermented kelp products to offer interesting possibilities for development of other types of kelp foods. Our prior research found no significant differences in consumer liking of sugar kelp sauerkraut-style products made with raw kelp plus 25% or 50% cabbage [23]. Due to the similarity of fermented kelp to sauerkraut, they will be referred to as “kelp- or kelp/cabbage sauerkraut” in this paper. The consumer liking of kelp sauerkraut formulated with blanched and/or previously frozen product is unknown. Therefore, the second objective of this study was to evaluate the effects of blanching and freezing of sugar kelp on the microbial quality, physical properties, and consumer acceptability of sauerkraut containing sugar kelp. A 50% kelp/cabbage sauerkraut blend was chosen for this study and was compared to a lab-made 100% cabbage sauerkraut. Findings are of economic significance to the seaweed industry as growers and processors attempt to diversify products and increase profit.

## 2. Material and Methods

### 2.1. Sample Preparation

Fresh sugar kelp (*Saccharina latissima*) was received on two different occasions in a space of three weeks in April 2019 for the two experiments (kelp salad and sauerkraut studies). Fresh, cultivated sugar kelp received from Maine Sea Farms (South Bristol, ME) was washed with tap water to remove debris and shredded with a food processor (RobotCoupe^®^, CL 50 Series E, Jackson, MS, USA) fitted with a 0.32 cm slicing disc. In both experiments, about 350 g of shredded kelp were weighed into 30.48 cm × 30.48 cm plastic bags (UltraSource, Kansas, MO, USA) and vacuum packaged (KOCH Ultravac, Model UV550, Wichita, KS, USA). Vacuum-packed bags of kelp were placed in a metal strainer and submerged in boiling tap water (100 °C) of about ¾ of a 50 L steam jacketed kettle for a prescribed time according to the experimental design. After blanching, the sample bags were immediately cooled in an ice/water slurry (~1 °C) for 1 min.

#### 2.1.1. Kelp Salad Study

**Figure 1:**
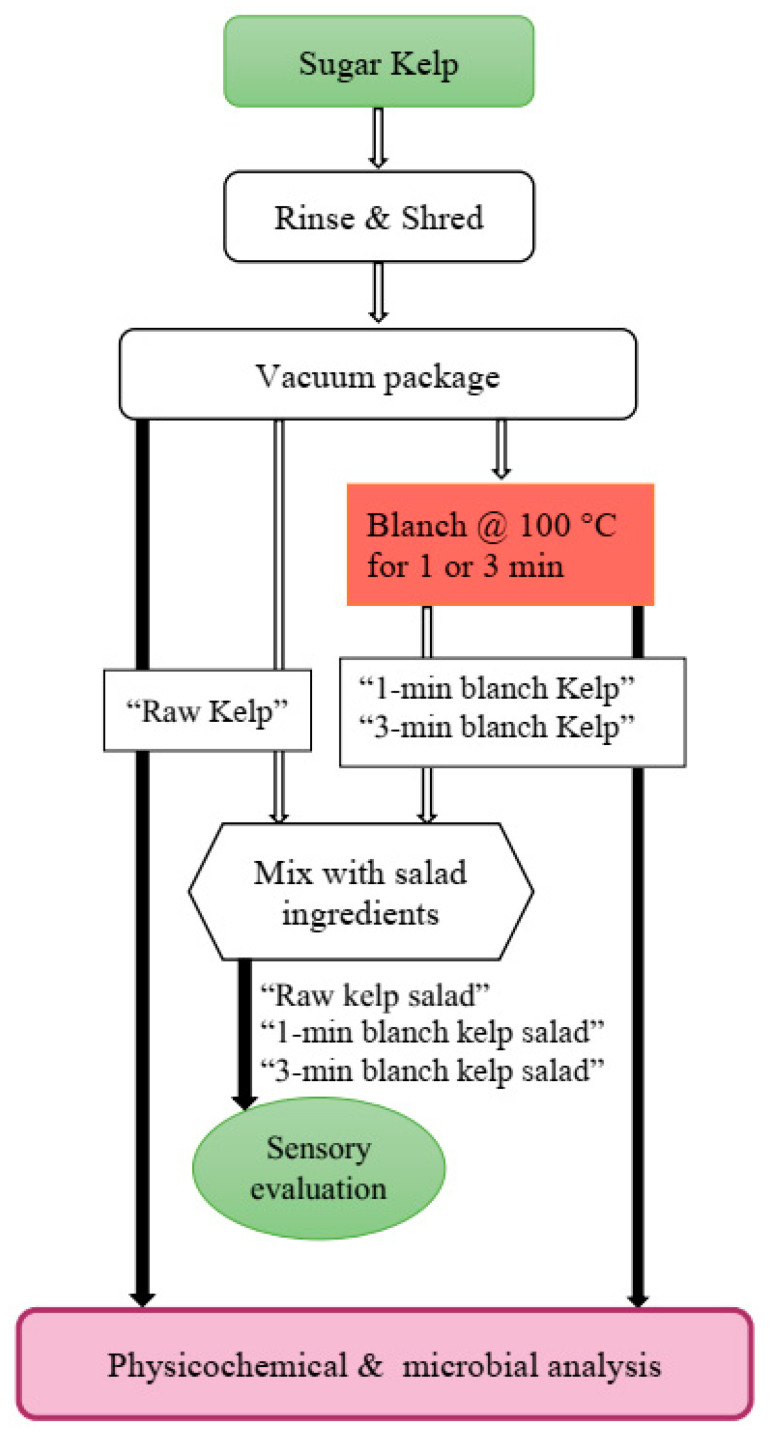
Flow diagram for kelp salad study.

Kelp was separated into three groups: a 1 min blanched, 3 min blanched, and unblanched (control) treatments. Blanching temperature, blanching time and vacuum packaging were based on the relatively higher product quality recommended by a previous study in our laboratory [25]. Random samples were aseptically taken from the vacuumed bags after blanching and analyzed in triplicate for physicochemical and microbial quality (Figure 1). The remaining replicates of each treatment were mixed together separately. A seaweed salad recipe from Food.com [26] was modified for this purpose. The samples were then processed into a seaweed salad for sensory evaluation. Shredded kelp from the three previously processed treatments were mixed with shredded carrots (1.3% salad weight) and sesame seeds (10.1%), before adding 0.15% of commercial Asian balsamic vinaigrette (containing balsamic vinegar, vegetable oil (soybean and/or canola), extra virgin olive oil, salt, garlic, spice, onion, xanthan gum, red bell pepper, mustard flour) (Ken’s Lite Balsamic Vinaigrette, MA, USA). Three salad treatments (blanched for 1 min or 3 min, raw) were prepared to evaluate the effects of blanching treatment on the consumer acceptability of the kelp (Figure 1).

#### 2.1.2. Kelp Sauerkraut Study

**Figure 2:**
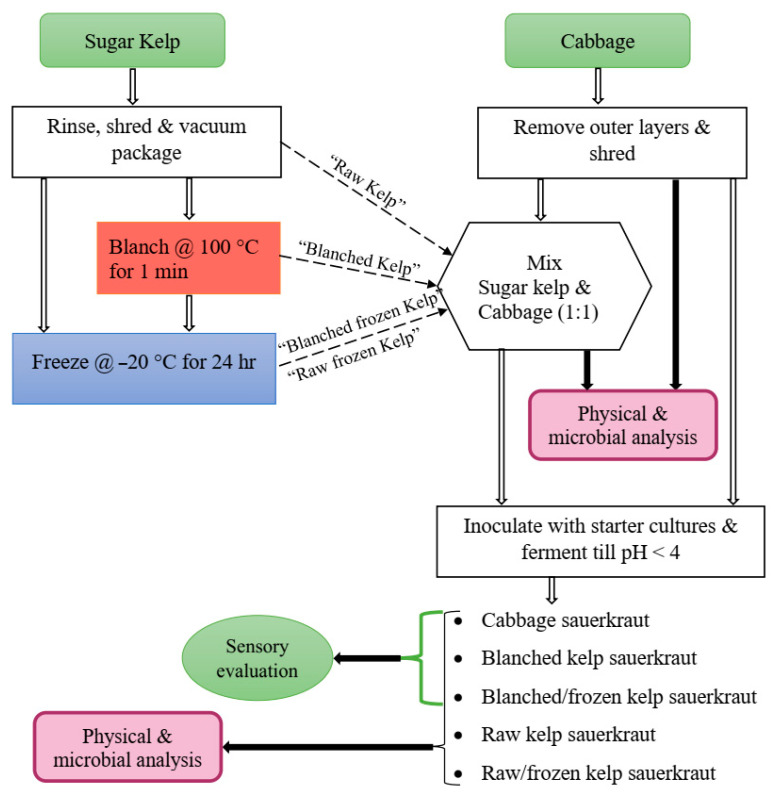
Flow diagram for kelp sauerkraut study.

The kelp sauerkraut study was designed to test for the effect of blanching and freezing on physicochemical and microbial properties of sugar kelp, which was developed into a value-added food product (kelp sauerkraut). The shredded kelp was divided into four treatments: raw, raw/frozen (−20 °C, 24 h), blanched (100 °C, 1 min), or blanched/frozen. Specifically, one of the blanched treatments (blanched/frozen) was immediately blast frozen after blanching, together with one of the raw kelp treatments (raw/frozen) at −30 °C (Southeast Cooler, Lithia Springs, GA, USA) for an hour, and then stored at −20 °C for 24 h before further processing. White cabbage (*Brassica oleracea*) was purchased from a local grocer. The outer leaves of cabbage were discarded, and the rest were washed and shredded with the same food processor used for shredding kelp. The four kelp treatments were combined with shredded cabbage (50% ratio) and manually mixed with kosher salt (2% of kelp/cabbage mix weight, Morton coarse Kosher salt, Chicago, IL, USA) for 5 min to produce a brine solution (Figure 2). The last treatment was 100% cabbage with 2% kosher salt, which served as a control. Each of the five treatments was packed into 3.785 L glass fermentation jars (Kombucha Brooklyn, Kingston, NY, USA) with a plastic lid and airlock. Treatments were subsequently inoculated aseptically in triplicate with starter cultures (see below) to ferment at ambient temperature (~22 °C) until a pH < 4.0 was achieved (an average of six days for all cabbage sauerkraut and nine days for kelp-containing sauerkrauts), at which time sauerkrauts were placed in storage at 4 °C for further analysis and sensory evaluation.

#### 2.1.3. Starter Culture Preparation

*Lactobacillus plantarum* (ATCC 8014) and *L. mesenteroides* subsp. *cremoris* were obtained from Microbiologics (St. Cloud, MN, USA) and DuPont (Danisco, Paris, France), respectively. Cultures were stored at −80 °C before use. The cultures were streaked separately onto *Lactobacilli* MRS agar (Alpha Biosciences, Baltimore, MD, USA) and placed into a 30 °C incubator for 48 h. One single colony of each culture was aseptically transferred into 9 mL of room temperature *Lactobacilli* MRS broth (Alpha Biosciences, Baltimore, MD, USA) and incubated at 30 °C for 24 h to achieve a population of ~9 log CFU/g for both cultures, verified by direct plating, which was used to inoculate the five treatments to achieve a target concentration of 10^1^ CFU/g for *L. mesenteroides* and 10^6^ CFU/g for *L. plantarum.*

### 2.2. Physicochemical Analyses

#### 2.2.1. Colorimetric Analyses

Color change in sample treatments was measured with a colorimeter (LabScan XE, Hunter Labs, USA) fitted with a 5.1 cm diameter aperture, which was standardized with white and black tiles. Sample shreds were placed to cover the bottom of a transparent cup and Hunter L*, a*, b* values were determined. Ten readings were recorded for each treatment replicate. Color change (ΔE) after processing was calculated in comparison to raw values using the following formula:

$${\Delta E}_{\mathrm{ab}}^{*}=\sqrt{{\left(L_{2}^{*}-L_{1}^{*}\right)}^{2}+{\left(a_{2}^{*}-a_{1}^{*}\right)}^{2}+{\left(b_{2}^{*}-b_{1}^{*}\right)}^{2}}$$

where L* denotes lightness using a scale from black (0) to white (100), a* denotes the red (+a) to green (−a) color axis, and b* denotes the yellow (+b) to blue (−b) color axis. For the kelp salad study, the subscript 1 represents color values for raw samples before blanching and 2 represents color values after blanching.

#### 2.2.2. Instrumental Texture

Texture analysis for all treatments was conducted using the Kramer shear method. Briefly, 10–15 g of shredded sample were loaded into a mini Kramer shear cell (TA-XTi2, Texture Technologies Inc., Scarsdale, NY, USA) with five flat blades set to travel 5 cm in a downward direction at 2 mm/s. Force (N) required to shear the sample was recorded as the firmness of the shredded kelp. Ten subsamples from each treatment replicate were analyzed, and values were averaged.

#### 2.2.3. Moisture Content

Moisture content (%) was determined using a convection oven (VWR International, Radnor, PA). Each treatment replicate was evaluated in duplicate, and values were averaged in percentage on a wet weight basis (wwb). Briefly, homogenized kelp samples (5 ± 0.002 g) in a pre-weighed aluminum pan were dried at 105 °C for 6 h (AOAC, Method 950.46) [27]. Pans containing the dried samples were re-weighed and the percent moisture was calculated.

#### 2.2.4. Total Phenolic Content (TPC) and Antioxidant Analysis

Blanched and raw samples used for salad were freeze-dried (VirTis Ultra, Warminster, PA, USA), ground, and extracted for analysis as previously described by Rajauria [28] with slight modifications. Freeze-dried samples (2 g) were mixed with 20 mL of 60% methanol (*v*/*v*) and shaken on a lab plate shaker at 210 rpm for 24 h at room temperature. The mixture was centrifuged at 2100× *g* for 10 min. All supernatants from the extraction and pellet wash (2 times) were collected and then brought to a final volume of 50 mL with deionized water. The extracts were stored at −20 °C prior to conducting total phenolic content (TPC) and ferric reducing antioxidant power (FRAP) assays.

Total phenolics were determined in duplicate using the Folin–Ciocalteau reagent. Absorbance was measured at 725 nm against a 42% methanol blank. Total phenol content was expressed as mg of gallic acid equivalent (GAE) per g of freeze-dried sample based on a gallic acid reference curve (0–200 ug/mL) [28].

The assay for ferric reducing antioxidant power (FRAP) procedure was conducted according to the method described by Rajauria [28]. Fe^3+^ in the FRAP reagent, which included 2,4,6-tripyridy-s-triazine (TPTZ), was reduced in the presence of the sample extracts, and a colored TPTZ–Fe^2+^ complex was formed. After 4 min, sample absorbance was measured at 595 nm against a deionized water sample blank. A standard curve was derived from the absorbances of 50–750 μM ferrous sulfate (FeSO_4_·7H_2_O) in deionized water. All samples were analyzed in duplicate and results were expressed as μmol ferrous sulfate equivalents (FSE) per gram of freeze-dried sample.

### 2.3. Determination of Microbiological Quality

In the kelp salad study, microbial safety analysis was performed on the raw control and blanched kelp treatments before incorporating them into salads. In the second study, samples were tested before and after fermentation of the five treatments. The presence of *Vibrio* spp., *Listeria monocytogenes*, *Salmonella* spp., and *Staphylococcus aureus* was assessed as described by FDA’s Bacteriological Analytical Manual [29]. Briefly, 25 g of each of the samples were placed aseptically into 225 mL of alkaline peptone water (28 °C) for *Vibrio*, Listeria enrichment broth (28 °C) for *Listeria*, lactose broth (35 °C) for *Salmonella* and tryptic soy broth with 10% NaCl and 1% sodium pyruvate (35 °C) for *S. aureus* in a stomacher bag and homogenized for two minutes using a BAGMixer 400 (Model P, Spiral Biotech, Advanced Instruments, Norwood, MA, USA). Afterward, the stomacher bag was incubated for 24 h and samples were plated (0.1 mL) on Thiosulfate–citrate–bile salts–sucrose agar (28 °C, *Vibrio*), Modified oxford agar (28 °C, *Listeria*), Xylose lysine deoxycholate agar (35 °C, *Salmonella*) and Baird–Parker (35 °C, *S. aureus*) in duplicate and incubated for 48 h for each of the treatment replicates. The presence of colony growth with expected morphology denoted the presumptive presence of pathogens.

To assess microbial quality, duplicate samples (10 g) of all treatment replicates in both experiments were mixed with 0.1% peptone and agitated for 2 min. After agitation, the samples were serially diluted in 0.1% peptone and spread plated onto tryptic soy agar (TSA) (Alpha Biosciences, Baltimore, MD, USA) and acidified potato dextrose agar (APDA, Alpha Biosciences, Baltimore, MD, USA) for aerobic plate counts (APC) and fungi, respectively. Plates were incubated at 37 °C for 48 h (TSA), and at room temperature for 5 days (APDA). Microbial populations were determined in log CFU/g for APC and fungi.

### 2.4. Sensory Evaluation

**Figure 3:**
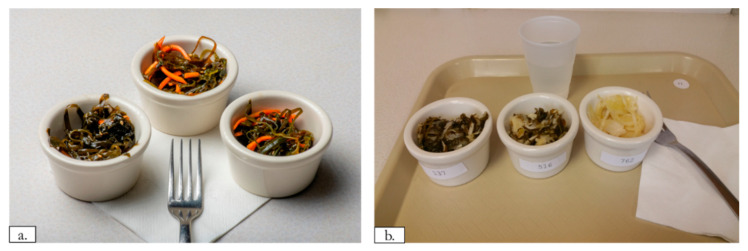
(**a**) Sugar kelp salad; (**b**) sugar kelp and/or cabbage sauerkraut.

This research was approved by the University of Maine Institutional Review Board for the protection of human subjects. All research participants provided their informed consent. In the kelp salad study, sensory evaluation was conducted to determine the effects of two blanching times on consumer acceptance of salad made from blanched or raw kelp One hundred and two sensory panelists (at least 18 years old) in the greater Orono, ME area interested in seaweed and not allergic to seaweed or the other salad ingredients were recruited via email and flyer notices to assess the acceptability of sugar kelp salad. Each of the three salads was kept at 5–10 °C in a covered aluminum dish before being served. Panelists were simultaneously presented with three 30 g samples of three kelp salads for evaluation (Figure 3a).

In the kelp sauerkraut study, 30 g of sauerkraut prepared as described previously was served for each of the three treatments: blanched kelp sauerkraut, blanched/frozen kelp sauerkraut, and the raw cabbage sauerkraut control. Eighty sensory panelists (older than 18 years) interested in consuming seaweed and sauerkraut were recruited via email and flyer notices to assess the acceptability of kelp and/or cabbage sauerkraut. Each treatment was kept at 5–10 °C in a covered aluminum dish prior to being served (Figure 3b).

For both studies, panelists were seated in individual booths with a combination of fluorescent and incandescent lighting at the Sensory Evaluation Center at the University of Maine. The three products were labeled with 3-digit random codes and were served in a ceramic ramekin with small cups of ~4 °C Poland spring water alongside. Sample order was randomized in each study to reduce the effects of flavor carry-over and order bias. Panelists were instructed to evaluate the samples, take a sip of water before testing each sample, and rate the acceptance of specific sensory attributes of the samples. A 9-point hedonic scale (from 1 = “Dislike Extremely” to 9 = “Like Extremely,” with 5 = “Neither Like nor Dislike”) was used to assess the acceptability of appearance, color, flavor, texture, and overall liking of samples [30] and a 5-point Just-About-Right (JAR) scale (1 = Not Firm/Tender, 2 = Somewhat Firm/Tender, 3 = Just About Right, 4 = Somewhat Too Firm/Tender, and 5 = Much Too Firm/Tender) was used to examine specific texture attributes (firmness and tenderness) for salad only [31]. Penalty analysis was performed for scores that were not JAR. Participants were asked to answer a set of questions relating to demographic characteristics, seaweed consumption habits, and attitudes towards consuming seaweed in both studies prior to consuming samples. Panelists were also asked if they would like to consume raw seaweed in the kelp salad study prior to consuming samples. Panelist were asked to select one descriptor that best described each salad treatment from a short list (chewy, firm, tender, juicy, mushy, soft, tough) based on previous research [32,33]. Additionally, panelists choose which forms they consume seaweed (as part of other foods like sushi, salad, soup, frozen smoothie cubes or in other form). In the kelp sauerkraut study, participants were additionally asked to check all that apply (CATA) for words that best described each sauerkraut sample after consumption. Panelists were asked to provide comments about the three treatments at the end of both studies. The test randomizations, experimental designs, and analyses were executed using SIMS 2000 (Sensory Computer Systems, Berkeley Heights, NJ, USA) software.

### 2.5. Statistical Analysis

Data from physicochemical, microbial, and sensory tests were analyzed using SPSS 20 (IBM, Armonk, NY, USA) at a significance level of *p* ≤ 0.05. One-way analysis of variance (ANOVA) was used to assess all one-level (treatment) effects. Multiway ANOVA was used to assess salad type and consumption frequency. Separation of treatment means was accomplished using Tukey’s honest significant difference (HSD) post hoc test. Pearson’s correlation was performed to evaluate correlations among variables. An independent t-test was used to compare the changes in color between the two blanched treatments in study one, and a pairwise t-test was used to compare kelp/cabbage qualities in treatments before and after fermentation in study two. A Cochran–Mantel–Haenszel test was used to determine whether JAR score distributions were different among the three products for firmness and tenderness attributes.

## 3. Results

### 3.1. Color

For the kelp salad study, blanching treatments significantly affected (*p* ≤ 0.05) the color of sugar kelp irrespective of the blanching time (Table 1). The L* and b* values increased while the a* values decreased when blanched. The difference in color between the raw kelp (control) and blanched kelp (∆E value) was visible as a change from golden brown to a vivid bright green color.

Regarding the kelp sauerkraut study, blanching and freezing of the kelp had no significant effects on a* and b* values of the four kelp/cabbage mix treatments prior to fermentation into kelp sauerkraut. Similarly, blanched sauerkraut treatments had no significant effect on a* and b* values as compared to raw treatments after fermentation. Kelp blanching resulted in significantly higher L* values in blanched kelp/cabbage mix as compared to raw/frozen kelp/cabbage mix prior to fermentation, but this difference was no longer observable after completion of fermentation (Table 2). Additionally, freezing was associated with decreased L* values among raw treatments after fermentation (Table 2).

### 3.2. Instrumental Texture

The textural parameter determined in kelp samples was shear force (Firmness, N). Blanching decreased kelp firmness, especially when blanching time increased from 1 to 3 min (Table 1). Kelp was blanched and/or frozen before mixing with cabbage prior to fermentation. For the kelp/cabbage mix prior to fermentation, blanching significantly decreased (*p* = 0.00, F-statistic = 152.86) firmness in both blanched, as compared to raw, treatments but freezing significantly decreased firmness in only raw treatments (Table 2). After fermentation, freezing had no impact on kelp sauerkraut treatments but blanching significantly reduced (*p* = 0.00, F-statistic = 115.94) firmness in kelp sauerkraut as compared to raw treatments. When comparing the firmness of each treatment pre- and post-fermentation, only the 100% cabbage control significantly decreased (Table 2).

### 3.3. Chemical Properties

Blanching had a significant impact on moisture content, which ranged from 86.3 to 91.5% (wwb). The longer blanching time resulted in significantly higher moisture content as compared to raw kelp (Table 3).

No significant trends in TPC and FRAP values were observed based on the blanching time (Table 3).

### 3.4. Microbiological Quality

Considering the kelp salad study, raw samples were compared to blanched samples with emphasis on the effects of blanching time on microbial quality. There were no significant differences in APC or fungi counts among raw, 1 min and 3 min blanching time samples, which were below 3 log CFU/g and 2.5 log CFU/g, respectively (Table 4). None of the pathogens tested (*Vibrio* spp., *Listeria monocytogenes*, *Salmonella* spp., and *Staphylococcus aureus*) were detected in any of the samples.

In the kelp sauerkraut study, APC and fungi counts before fermentation ranged from 2.0 to 2.4 log CFU/g (Table 4). Blanching and freezing had no impact on APC or fungi counts. When comparing the APC and fungi counts in the different treatments before and after fermentation, only raw kelp sauerkraut had a significant increase in the fungi population after fermentation. While not measured in this study, previous work [23] has shown that levels of lactic acid bacteria are closely negatively correlated with pH, and so are expected to have increased proportionally during fermentation. A presumptive positive result for *Vibrio* sp. was detected in one replicate of the raw kelp/cabbage mix samples but was not detected after fermentation.

### 3.5. Sensory Evaluation

#### 3.5.1. Demographics and Consumption Trends

Demographic and consumption habit questions were asked before the evaluation of the salads. More females (64%) took part in the evaluation (Table 5). The majority (72.5%) of the sensory participants for the kelp salad evaluation were 35 years old or younger. Sixteen participants were Asian, and 78 were white.

The participants indicated that seaweed was consumed more at restaurants than at home. Results showed that 64.7% of participants eat seaweed raw, 74.5% of participants consume it as part of other food like sushi, 44.1% as salad, 35.3% as soup, and the remainder in other forms, including frozen kelp smoothie cubes. More than half of the panelists (61.8%) chose flavor as the most important seaweed characteristic and color as the least (<1%). Additionally, 87.2% of participants indicated a willingness to buy a 113.4 g (4 oz.) bowl of seaweed salad for a USD 2–4 price range (Table 6).

Sixty percent of the participants in the kelp sauerkraut study were female and 70% of participants were younger than 35 years of age (Table 5). More than half of the participants were white (~63%) and about 29% were Asian. About 41% of participants claimed to consume seaweed 1–6 times a year, and 30% reported consuming 1–2 times a month. Over 75% of participants knew that fermented foods, such as sauerkraut, may contain probiotics that are associated with disease prevention and improved digestion; 48.8% of panelists reported consuming probiotics as either a food or dietary supplement ≥ 1 time per week (Table 6).

#### 3.5.2. Sensory Attributes

The mean acceptability scores for five sensory attributes (appearance, color, flavor, texture, and overall liking) of the kelp salad ranged from 5.4 to 6.7 on the 9-point hedonic scale which were between “neither like nor dislike” and “like moderately” (Table 7). Generally, the blanched samples used to prepare kelp salad were liked more than the raw sample for color, flavor, and overall liking (Table 7). No significant differences were seen in any sensory attributes between the blanched treatments. Overall acceptability scores for all three treatments had strong, significant (*p* ≤ 0.01) positive correlations with texture (r = 0.67) and flavor sensory scores (r = 0.91). Notably, frequent (at least 2–3 times a month) consumers of seaweed and those that normally consume seaweed at restaurants rated the 3 min blanched kelp salad significantly higher than the 1 min and raw kelp salad for “overall liking”.

Among all salad treatments, “chewy” and “firm” were the CATA descriptors selected most frequently to describe the characteristics of the three kelp salad treatments (Table 8). Assessment of descriptors did not significantly differ (*p* > 0.05) when compared with the other treatments using a chi-squared test.

**Figure 4:**
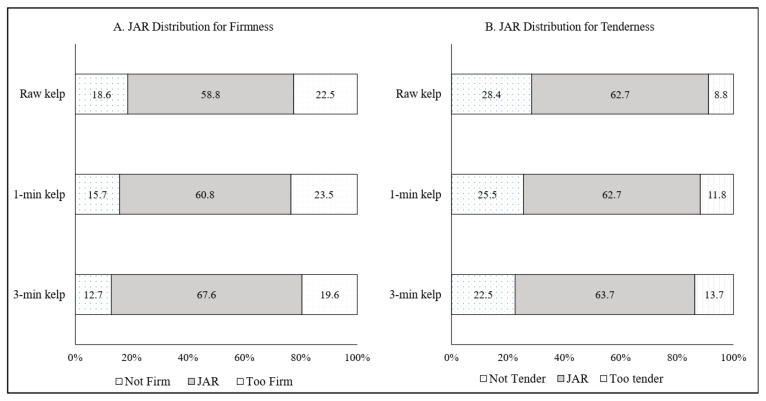
Just-About-Right (JAR) categorical scores (n = 102 consumers) for (**A**) firmness and (**B**) tenderness for raw kelp (control), 1 min blanched kelp, and 3 min blanched kelp salad.

The subsequent JAR analysis focused on the specific texture attributes “firmness” and “tenderness,” and whether consumers considered them to be ideal. Results from JAR analysis among the salad treatments are shown in Figure 4. For an attribute to be considered ideal, at least 70% of the responses should be “Just About Right” [31]. Above 20% of respondents judged all three salad products to be too firm and not tender, and none of the attributes had the right degree of firmness and tenderness as JAR did not reach the ideal 70% mark (Figure 4). The Cochran–Mantel–Haenszel test showed no statistically significant differences among the three products in the distributions of the assessors’ scores on the JAR scale for the firmness (*p* > 0.05; 0.698) and tenderness (*p* > 0.05; 0.776) attributes.

**Figure 5:**
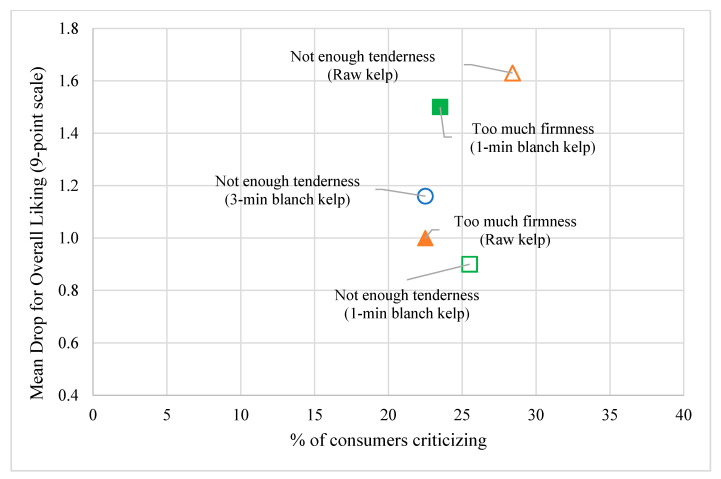
Mean drops (penalties) in overall liking on a 9-point hedonic scale (n = 102 consumers) from penalty analysis corresponding to the scale ends for each JAR texture attribute of firmness and tenderness for raw kelp, 1 min blanch kelp, and 3 min blanch kelp salad samples.

Penalty analyses of the raw kelp, 1 min blanched kelp, and 3 min blanched kelp salad samples were performed to determine whether respondents’ ratings for firmness and tenderness which were not JAR (less than 70% of responses were JAR) were associated with a mean drop in hedonic ratings of the Overall liking (Figure 5). Mean drops of 1.5–1.9 are concerning, drops of 1–1.49 are slightly concerning, and 0–0.99 are very slightly concerning [30,31]. Raw kelp and 3 min blanched kelp salad samples received concerning penalties for “Not enough tenderness,” while 1 min blanched kelp salad samples received concerning penalties for “Too much firmness”. These mean drops reflected on the “overall liking” mean hedonic scores of raw kelp salad (5.7 ± 1.7), 1 min blanched kelp (6.5 ± 1.7), and 3 min blanched kelp salad samples (6.5 ± 1.7).

Only blanched (as opposed to raw) kelp was used for sensory evaluation of kelp sauerkrauts in the kelp sauerkraut study based on the more positive results for blanched samples obtained in the kelp salad study. The mean acceptability scores for the control cabbage sauerkraut were higher for flavor and overall liking than for the blanched and blanched/frozen kelp sauerkrauts (Table 9). There were no differences among samples for appearance, color, and texture. The aroma of the blanched kelp sauerkraut had a lower mean hedonic rating than the sauerkraut with cabbage alone. Liking of blanched kelp sauerkraut was not significantly different from blanched/frozen sauerkraut for all sensory attributes. Overall acceptability scores for all sauerkraut treatments had strong, significant (*p* ≤ 0.01) positive correlations with texture (r = 0.63), aroma (r = 0.64), and flavor scores (r = 0.90). Focusing on kelp sauerkraut only, overall acceptability scores had a significant (*p* ≤ 0.01) moderate positive correlation with texture (r = 0.61), and aroma (0.61), and strong, significant (*p* ≤ 0.01) positive correlations with flavor scores (r = 0.91). The study showed no significant differences in “overall liking” scores between low (<time a year) and high (≥1 time a month) frequency consumers of sauerkraut. High frequency consumers rated the blanched kelp sauerkraut (6.5) and blanched/frozen kelp sauerkraut (6.7) higher than the less frequent consumers of sauerkraut (both kelp treatments = 5.8).

The majority of panelists described all sauerkraut treatments (raw cabbage-, blanched kelp- and blanched frozen-sauerkraut) as “crunchy,” and “pickled” (Table 10). Assessment of descriptors using chi-squared indicated significant differences (*p* ≤ 0.05) among treatments. Cramer’s V coefficient (0.243) indicates that sauerkraut treatment had a small to medium effect on sauerkraut descriptors [34]. Interestingly, ≥25% of panelists described all treatments as fresh and kelp sauerkraut as having ocean breeze flavor. Notably, panelists described blanched fresh kelp sauerkraut as “pungent” as compared to blanched/frozen sauerkraut, whereas as “well-rounded product” was used to describe blanched/frozen sauerkraut as compared to blanched fresh sauerkraut. A few panelists described the sauerkraut treatments in the comment section as “looks bright and smells good,” “color was more interesting in seaweed sauerkraut than cabbage only,” and “very acidic”.

## 4. Discussion

### 4.1. Physicochemical Properties

Color is an important index for the quality of processed sugar kelp. The golden-brown color of kelp immediately transformed to a green color when blanched, similar to the color change of kelp when blanched in other studies [19,24]. The high intensity of greenness seen in blanched kelp indicates a breakdown of the brown pigment fucoxanthin [35], which masks the green color of chlorophyll in raw kelp. The longer blanching time (3 min) at 100 °C resulted in a lower green intensity as compared to the shorter blanching time (1 min). A longer exposure to heat likely led to the formation of chlorophyll breakdown products including the brownish pigment pheophytin and the yellow brown olive pigment pyropheophytin, as a result of the replacement of the central magnesium atom with a hydrogen atom [36,37]. The trend was similar to the green color, expressed as -a*/b*, of blanched winged kelp (*A. esculenta*) but contrary to that of sugar kelp samples, when they were subjected to various blanching temperatures (60–95 °C) and times (1 s–60 min). Sugar kelp showed an upward trend of green color intensity [19]. Hunter a* value had a mildly inverse correlation (*p* ≤ 0.0001, r = −0.389) with the overall liking hedonic score of kelp salad, with the inverse of a* indicating the intensity of kelp greenness. These results highlight the need for strict control of blanching procedures to maximize consumer acceptability.

Kelp firmness decreased as blanching time progressed, suggesting a thermal breakdown of polysaccharides in kelp cell walls. Kelp polysaccharides are comprised mainly of alginate that consists of unbranched chains of contiguous β-l,4-1inked D-mannuronic acid blocks, and blocks of contiguous α-l,4-1inked L-guluronic acid [38,39], which become porous when heated. The increase in moisture content after blanching may have been due to the abundant kelp polysaccharides absorbing and retaining some of the water molecules which would have been lost to dripping in a raw product [40,41,42]. There is a possibility that the increase in moisture content may result in increased profits for kelp processors since finished products are sold by weight. Blanching slightly decreased total phenolic contents (TPC), and antioxidant capacity as determined by the FRAP method. The observed low values of TPC and FRAP in all kelp salad treatments may be as a result of shredding as seen in our previous shredded frozen kelp study [25]. Although no significant differences in TPC or FRAP values were found among treatments, the slight decline in TPC and FRAP values as blanching time increased suggests a negative impact of thermal treatment in preserving phenolic compounds and antioxidant capacity in sugar kelp, as expected. TPC values in the present study for fresh and blanched kelp treatments (Table 3) are below the range for fresh and blanched sugar kelp (2.4–54.4 mg·GAE/g [43]) and within the range of fresh harvested sugar kelp in different seasons (0.84–2.41 mg·GAE/g [44]) reported in different studies. FRAP values were within the range of total antioxidant capacity (TAC) in fresh harvested sugar kelp in different seasons (0.84–2.41 mg·GAE/g DM [44]).

Overall, blanching may aid in commercializing kelp products because it increased the moisture, lightness, and greenness of kelp, which positively impacted sensory scores. The optimal texture preferences of consumers should be defined in future research.

L*, a*, and b* values for kelp sauerkraut (Table 2) were similar to those of 50% sugar kelp sauerkraut-style product reported in the literature [23]. There were no significant differences between the raw and blanched kelp/cabbage mix for a* and b* values, possibly due to the mixture of the white cabbage. Similarly, there was no significant change in color for b* values (indicating yellowness) between raw kelp sauerkraut and blanched kelp sauerkraut. A previous study also reported no change in the visual appearance descriptor (yellow–green) between fresh kelp and fermented kelp when subjected to a descriptive sensory test by 13 panelists [24]. The range of firmness values for kelp/cabbage sauerkraut in our study (Table 2) was higher than for fermented kelp/cabbage sauerkraut stored at 3 °C for 60 days post inoculation (<150 N) [23]. This indicates that sauerkraut firmness may have decreased as fermentation progressed during low-temperature storage. When comparing products prepared from blanched fresh vs. blanched/frozen kelp, freezing did not have a significant immediate effect on the color or firmness of the kelp sauerkraut. Thus, freezing may provide seaweed producers with an alternative to prolong the shelf-life of sugar kelp for subsequent food production. Similarly, the firmness of frozen blanched sugar kelp remained unchanged during six months of frozen storage in a previous study conducted in our laboratory [25]. It would be valuable to see whether longer-term frozen storage of the kelp (e.g., 1 year) would impact subsequently prepared sauerkraut texture.

### 4.2. Microbiological Analysis

Aerobic plate count (APC) and fungi counts were low in both experiments, suggesting a minimal risk of kelp salad and kelp sauerkraut spoilage from microorganisms. The results were similar to previously reported microbial populations (between 1 and 3 log CFU/g) of *A. esculenta* and *S. latissima* when subjected to different heat treatments [19]. Blanching significantly reduces microflora in vegetables, where either below or near the detection level (1 log CFU/g) reduction was observed in *Enterobacteriaceae*, total yeast, and mold counts [18]. A similar reduction in APC and fungi counts of kelp was observed in both experiments after blanching; however, the reductions were not significant. For sauerkraut, Khanna [45] reported similar fungi count range (~2.5 log CFU/g) and higher APC range (3.9–4.6 log CFU/g) in cabbage sauerkraut as compared to our study. About 8 log CFU/g of APC was observed in another cabbage sauerkraut study after two days of fermentation, which had a slight but not significant reduction in APC as fermentation progressed for 37 days [46]. As initial levels of APC in this study were extremely low, it is not surprising that a significant decrease attributable to fermentation was not observed. When cabbage was mixed with kelp, about a 23% increase in APC was observed when different ratios of kelp/cabbage mixture were fermented into sauerkraut in a different study, and levels of lactic acid bacteria were negatively correlated with pH [23]. The impact of fermentation on microflora (APC) in cabbage and/or kelp sauerkraut in the kelp sauerkraut study was not significant except in one treatment (Table 4).

Based on the numerous microbial pathogens and toxins found in the marine environment that are linked to human diseases [47] and potential cross contamination during post-harvest processing of seaweed [48], there is a possibility of harborage of pathogens on sugar kelp during production and processing. Water temperatures in the marine environment where seaweed is grown are increasing and these high temperatures are associated with elevations of *Vibrio* populations [49]. Moreover, there have been outbreaks of salmonellosis, listeriosis and *Staphylococcus aureus* poisoning associated with minimally processed or ready to eat vegetables via contaminations [50,51,52]. Therefore, seaweed could be contaminated if not handled properly. The presence of *S. aureus, Salmonella, Listeria monocytogenes*, and *Vibrio* was assessed in all treatments to ensure food safety. However, the absence of these pathogens in study one is encouraging for the marketability of fresh kelp. The detection of a presumptive *Vibrio* colony in one replicate of the raw (fresh) kelp/cabbage mix (before fermentation) sample suggests that the presence of *Vibrio* sp. on kelp should be expected to be sporadic since *Vibrio* sp. are common in the waters where kelp is grown. Interestingly, all samples of fully fermented sauerkrauts were negative for presumptive *Vibrio*. Results reinforce the knowledge that fermentation conditions, especially the decrease in pH, can inactivate pathogens in some fermented food products. Similarly, *Bacillus cereus* was absent in inoculated kelp after heat treatment and fermentation [23] and there was a reduction in pathogen growth as pH declined when cabbage was fermented with *Lactobacillus plantarum* [53] and *Leuconostoc mesenteroides* [54]. Moreover, several studies have reported the antimicrobial activity of seaweed, which is higher in brown seaweed extracts than red or green [27,29]. Exudates from kelp as a result of shredding may have released bacteriostatic compounds from this brown seaweed which could act against spoilage microorganisms and pathogens. However, an inoculation study is recommended to confirm whether the fermentation process can inactivate pathogens present in the kelp/cabbage products.

### 4.3. Sensory Evaluation

Generally, a mean liking score of ≥7 on a 9-point hedonic scale is associated with highly acceptable sensory quality [55]. The overall liking scores for sensory evaluation for the salad treatments (raw, 5.7; 1 min blanched, 6.5 and 3 min blanched frozen, 6.5) suggest that blanching had a positive impact on consumer acceptance of kelp. Since seaweed products are less popular in the West compared to Asian nations, it is important to note that the hedonic scores are promising because most of the panelists identified as white. The mean acceptability scores for color, texture, flavor, and overall liking of seaweed for all the salad treatments fell within the range of 5.5–6.7, which is approximately within the 6-point score comparable to the “like slightly” category. The large variation in “overall liking” for raw kelp salad (5.7 ± 1.4), 1 min blanched (6.4 ± 1.7) and 3 min blanched/frozen (6.5 ± 1.7) may be a result of many respondents (42.2%) being infrequent seaweed consumers (<1–2 times a year). A MANOVA analysis indicated the frequent consumption group (2–3 times a month to ≥ 1 in a week) rated the “overall liking” of raw, 1 min, and 3 min blanched kelp salad as 5.7, 6.3, 7.2, respectively. Three-minute blanched kelp salad was rated significantly higher than raw kelp for overall liking, suggesting that blanching time influenced how respondents familiar with seaweed products liked kelp salad. The relatively higher ratings of blanched kelp compared to raw kelp salad samples (Table 9) may be due to the noticeably juicy and tender nature described by sensory participants. As previously noted, this texture could be a result of the increase in moisture content in blanched kelp. However, participants did not deem blanched treatments or raw kelp salads to be ideal for texture (chewiness and tenderness) from the JAR analysis, possibly as a result of the heterogeneity of kelp products. Consumers were able to differentiate between the color of the two blanching treatments and raw samples, which strongly correlated with instrumental color analysis. The greenness of kelp after blanching correlated to the overall liking of salad and it could be that green represented a more familiar vegetable product because of consumers’ perceptions about the color green and nature [56]. In view of the high ratings for blanched kelp color, blanched products (kelp/cabbage sauerkraut) were selected as the focus for study two and they were compared to cabbage sauerkraut for sensory evaluation.

Scores from the sensory evaluation study of kelp sauerkraut suggest that fermentation could be used as an alternative method to produce seaweed foods for the consumer market. Although, over three-quarters of the panelists knew fermented foods such as sauerkraut had probiotics, it did not correspond to a higher sauerkraut or seaweed consumption. Moreover, familiarity with probiotics in fermented foods did not significant impact the sensory attribute “overall liking” among sauerkraut treatments (cabbage = 6.4 ± 1.7, blanched kelp = 6.4 ± 1.5, blanched frozen = 6.8 ± 1.6). Comments such as “looks bright and smells good” and “color was more interesting in seaweed sauerkraut than cabbage only,” among others, suggest that the bright colors of the sugar kelp mixed with cabbage were more appealing to some consumers than the pale color of cabbage only. However, no significant differences were recorded among treatments based on the hedonic color score means. Texture was the most highly rated attribute of all the sauerkrauts treatment compared to a previous seaweed sauerkraut study [23]. The majority of respondents claimed that all treatments were salty (Table 9), and this perception may have affected the overall liking of the products. Fermented kelp had a high rating (~9 on a 12-point scale) for salty taste when subjected to a descriptive sensory test by 13 panelists [24]. In the same way, kelp sauerkrauts were clearly described as saltier than the cabbage control sauerkraut, possibly due to the salty environment in which the kelps are grown. The general saltiness described by the panelists for all the treatments may also have been a result of the 2% NaCl used to produce sauerkraut. The amount of salt added was not adjusted for existing sodium content. Previous research reported that the use of a mineral salt with a low sodium chloride content (57% NaCl, 28% KCl, 12% MgSO_4_, 1% SiO_2_ and 2% lysine hydrochloride) resulted in a preferred milder tasting sauerkraut as compared to sauerkraut produced with ordinary salt [57]. Another study reported of a positive effect on the sensory quality of sauerkraut with 0.5% salt concentration as compared to 1.5%, 2.5% and 3.5% [58]. A lower added salt content in the sauerkraut treatments in this study may have increased “overall liking” scores, even beyond 7.1, 6.5, and 6.7 for cabbage, blanched fresh-, and blanched frozen kelp sauerkraut, respectively, by the more frequent consumers. The addition of kelp in kelp/cabbage sauerkraut significantly reduced the aroma and flavor liking scores as compared to cabbage sauerkraut. The lower responses of participants choosing descriptors such as “salty,” “pungent,” “sour,” “tangy,” “fishy,” “brackish,” and “ocean breeze” for blanched/frozen kelp sauerkraut as compared to blanched fresh sauerkraut could suggest that freezing of samples masked some of these notes of kelp. This is a good indication that freezing could be an alternative preservation method to drying seaweed to enhance product quality for consumers who prefer milder tasting kelp products. The mean acceptability score for kelp sauerkraut treatments (containing 50% cabbage) for “overall liking” was slightly below 7, which is equivalent to “like moderately,” and indicates promise for acceptance of kelp sauerkraut. The higher overall liking score of the cabbage sauerkraut control was likely due to flavor and aroma, and it suggests that future kelp sauerkraut optimization may be required to increase the sensory score for kelp sauerkraut (>7.0).

Value addition of seaweed, especially the development of food products appealing to U.S. consumers, will increase their familiarity with seaweed as a food. Such products should be created to increase revenue and satisfy consumers’ changing demands, which are driven by parameters such as population growth, lifestyle and economic changes, and increased awareness about healthy foods.

## 5. Conclusions

With the increase in production of seaweed in the West, data gathered from this research show that kelp could be utilized and consumed as vegetables by consumers. The study revealed that preservation processes had some positive impact on kelp quality and consumer acceptability. Blanching increased greenness but decreased firmness of kelp. Results from sensory acceptability tests indicate that consumers may like blanched kelp food products more than raw, possibly due to the color change and reduced firmness. Therefore, we can recommend minimal processes such as blanching and freezing of seaweed for extension of the short shelf life of fresh kelp. Use of such processes will extend marketable life of kelp and may allow preservation for use in formulated foods independent of harvest season. However, cost of water and energy should be considered. Moreover, the absence of pathogens after fermentation from the kelp sauerkraut study confirm that fermented foods are typically safe however, proper hygiene and sanitation practices should not be compromised to prevent possible cross-contamination from the environment during and after kelp sauerkraut production. Moreover, freezing can increase kelp retail availability throughout the year and also mask some aroma notes of kelp such as pungency and fishiness when used to develop products. Future studies are warranted to evaluate the impact of an extended frozen storage on value added kelp products, since this study focused on the immediate effect of freezing on kelp sauerkraut. Additionally, blanching, freezing and fermenting kelp into sauerkraut can increase the commercial availability of seaweed products and promote the development of diverse seaweed products that could be easily made at home or conveniently sold in the marketplace year-round. These findings have important implications for the growing U.S. seaweed industry for many economical and nutritional reasons.

## Figures and Tables

**Table 1:** Color (Hunter L*, a*, b*) and texture of raw and blanched treatments of sugar kelp for salad (mean ± SD (n = 3)).

Treatments	L*	a*	b*	∆E Value	Firmness (N)
Raw	15.3 ± 1.7 ^c^	3.88 ± 0.9 ^c^	13.8 ± 1.5 ^b^	--	280.2 ± 37.8 ^a^
Blanched for 1 min	19.2 ± 2.8 ^b^	−2.18 ± 0.9 ^a^	18.5 ± 2.1 ^a^	9.0 ± 1.8 ^a^	227.1 ± 57.4 ^b^
Blanched for 3 min	20.5 ± 1.5 ^a^	−1.04 ± 1.0 ^b^	17.8 ± 2.2 ^a^	8.3 ± 1.7 ^a^	182.3 ± 32.1 ^c^

One-way ANOVA except for ∆E values (independent *t*-test). Superscripts: different letters within column indicate significant differences among treatments (*p* ≤ 0.05). Hunter (L*, a*, b*): L* = lightness, a* = red/green, b* = yellow/blue, ∆E = change in color.

**Table 2:** Color (Hunter L*, a*, b*) and firmness of kelp and/or cabbage mix treatments before and after fermentation (mean ± SD (n = 3)).

Treatments	L*		a*		b*		Firmness (N)	
Fermentation	Before	After	Before	After	Before	After	Before	After
Cabbage only	67.2 ± 2.1 ^aA^	65.1 ± 1.0 ^aA^	0.64 ± 0.9 ^aA^	0.29 ± 0.7 ^cA^	27.6 ± 2.7 ^aA^	27.0 ± 1.2 ^aA^	274.4 ± 10.6 ^aB^	233.9 ± 15.1 ^aA^
Raw kelp and cabbage	40.2 ± 2.7 ^bcA^	42.6 ± 1.2 ^bA^	2.07 ± 1.1 ^aA^	2.07 ± 0.4 ^aA^	16.0 ± 1.4 ^bA^	16.9 ± 1.1 ^bA^	238.4 ± 14.2 ^bA^	225.4 ± 15.0 ^aA^
Raw/frozen kelp and cabbage	40.0 ± 1.6 ^cA^	38.7 ± 1.0 ^cA^	2.13 ± 1.2 ^aA^	2.11 ± 0.9 ^aA^	14.7 ± 1.0 ^bA^	15.7 ± 0.7 ^bA^	229.5 ± 16.1 ^bcA^	225.7 ± 15.1 ^aA^
Blanched kelp and cabbage	43.6 ± 1.9 ^bA^	40.6 ± 1.3 ^bcA^	1.97 ± 1.0 ^aA^	1.58 ± 0.8 ^abcA^	14.9 ± 0.8 ^bA^	15.8 ± 0.9 ^bA^	201.0 ± 12.3 ^cA^	188.5 ± 13.7 ^bA^
Blanched/frozen kelp and cabbage	44.0 ± 2.2 ^bA^	40.4 ± 1.1 ^bcB^	1.80 ± 1.6 ^aA^	1.84 ± 0.9 ^abA^	15.2 ± 1.0 ^bA^	16.5 ± 1.0 ^bA^	199.4 ± 14.5 ^cA^	198.1 ± 11.3 ^bA^

Before fermentation, samples were 50% kelp/cabbage mixture and samples were 50% kelp/cabbage sauerkraut after fermentation. One-way ANOVA among treatment (column); pairwise *t*-test before and after fermentation (row). Superscripts: different small letters indicate significant differences among treatments (within column); different capital letters indicate a significant difference before and after fermentation (within row). A probability level of 0.05 (*p* ≤ 0.05) was selected for significance. Hunter (L*, a*, b*): L* = lightness, a* = red/green, b* = yellow/blue.

**Table 3:** Chemical properties of raw and blanched treatments of sugar kelp for salad (mean ± SD (n = 3)).

Treatment	Moisture (%)	TPC (mg GAE/g)	FRAP (μmol FSE/g)
Raw	86.3 ± 5.0 ^b^	1.5 ± 0.7 ^a^	5.3 ± 1.6 ^a^
Blanched for 1 min	90.6 ± 0.8 ^ab^	1.1 ± 0.6 ^a^	3.6 ± 0.8 ^a^
Blanched for 3 min	91.5 ± 0.4 ^a^	0.8 ± 0.3 ^a^	3.9 ± 2.0 ^a^

TPC = Total phenolic content. FRAP = ferric reducing antioxidant power. TPC and FRAP are measured in grams of freeze-dried sample. Superscripts: different letters within columns indicate a significant difference among treatments (*p* ≤ 0.05).

**Table 4:** Enumeration of aerobic plate count and fungi of sugar kelp in the two experiments (mean ± SD (n = 3)).

Treatment	APC (Log CFU/g)		Fungi (Log CFU/g)	
Salad study				
Raw	2.9 ± 0.4 ^a^		2.1 ± 0.3 ^a^	
Blanched for 1 min	2.6 ± 0.2 ^a^		2.4 ± 0.5 ^a^	
Blanched for 3 min	2.4 ± 0.5 ^a^		2.2 ± 0.4 ^a^	
Sauerkraut study				
(fermentation)	Before	After	Before	After
Cabbage only	2.2 ± 1.0 ^aA^	2.2 ± 0.8 ^aA^	2.3 ± 1.7 ^aA^	2.2 ± 0.1 ^aA^
Raw kelp/cabbage	2.3 ± 1.1 ^aA^	2.1 ± 0.7 ^aA^	2.2 ± 1.3 ^aB^	2.5 ± 0.3 ^aA^
Raw frozen kelp/cabbage	2.3 ± 0.9 ^aA^	2.4 ± 0.5 ^aA^	2.0 ± 0.9 ^aA^	2.0 ± 0.2 ^aA^
Blanched kelp/cabbage	2.3 ± 0.6 ^aA^	2.2 ± 0.1 ^aA^	2.0 ± 0.8 ^aA^	2.1 ± 0.1 ^aA^
Blanched frozen kelp/cabbage	2.4 ± 1.0 ^aA^	2.1 ± 0.4 ^aA^	2.4 ± 0.1 ^aA^	2.4 ± 0.2 ^aA^

APC = Aerobic plate count. Before fermentation, samples were 50% kelp/cabbage mixture and samples were 50% kelp/cabbage sauerkraut after fermentation. One-way ANOVA among treatment; pairwise *t*-test before and after fermentation. Superscripts: different small letters indicate significant difference among treatments; different capital letters indicate significant difference before and after fermentation (*p* ≤ 0.05).

**Table 5:** Demographics of participants for kelp salad and sauerkraut sensory evaluation.

Parameters		Salad Study	Sauerkraut Study
		n = 102 (%)	n = 80 (%)
Gender	M	36 (35.3)	32 (40.0)
	F	65 (63.7)	48 (60.0)
	Did not answer	1 (1.0)	-
Age (years)	18–25 years	43 (42.2)	17 (21.2)
	26–35	31 (30.4)	39 (48.7)
	35–45	10 (9.8)	11 (13.8)
	46–55	7 (6.9)	5 (6.3)
	56+	11 (10.7)	8 (10.0)
Race	American Indian/Alaska Native	1 (0.9)	0 (0.0)
	Asian	16 (15.7)	23 (28.8)
	Black/African American	5 (5.0)	4 (5.0)
	White	78 (76.5)	50 (62.5)
	Prefer not to say	0 (0.0)	3 (3.7)
	Did not answer	2 (1.9)	-

**Table 6:** Responses of consumption behavior of participants for kelp salad and sauerkraut sensory evaluation.

Parameters		Salad Study	Sauerkraut Study
		n = 102 (%)	n = 80 (%)
Would you like to consume your seaweed raw?	Yes	66 (64.7)	N/A
	No	32 (35.3)	
Where do you usually consume seaweed?	Restaurant	58 (56.9)	
	Home	24 (23.5)	
	Other	8 (7.8)	N/A
	Not applicable	9 (8.8)	
	Did not answer	3 (2.9)	
Approximately how often do you consume seaweed?	<1 year	9 (8.8)	34 (42.9)
	1–2 times a year	34 (33.3)	N/A
	1–6 times/year	N/A	32 (40.0)
	1–2 times a month	17 (16.8)	11 (13.8)
	2–3 times a month	34 (33.3)	N/A
	Weekly	6 (5.9)	N/A
	>2 times a week	2 (1.9)	N/A
	Weekly or >1 time a week	N/A	3 (3.7)
What would make you consume seaweed more often? (CATA)	Availability	72 (70.6)	
	Ready-to-eat	53 (51.9)	
	Lower price	34 (33.3)	
	Sustainability	34 (33.3)	N/A
	Sold fresh	31 (30.4)	
	Minimally processed	26 (25.5)	
	Longer shelf-life	21 (20.6)	
What form of seaweed products do you typically consume?	As part of other foods like sushi	76 (74.5)	
	Salad	45 (44.1)	N/A
	Soup	36 (35.3)	
	Frozen smoothie cubes	2 (1.9)	
	Other forms	16 (15.7)	
Price for a ready-to-eat four-ounce (113.4 g) seaweed salad bowl?	Would not buy	8 (7.8)	
	USD 2.00	24 (23.5)	
	USD 3.00	41 (40.2)	N/A
	USD 4.00	24 (23.5)	
	USD 5.00	5 (5.0)	
Which sensory characteristic of seaweed is most important to you?	Aroma	6 (5.9)	
	Color	3 (2.9)	N/A
	Flavor	63 (61.8)	
	Texture	30 (29.4)	
Did you know that fermented foods, such as sauerkraut, contain probiotics?	Yes	N/A	19 (23.8)
	No		61(76.2)
How often do you eat foods or dietary supplements containing probiotics?	Less than once per year		5 (6.3)
	1–4 times per year		15 (18.7)
	1–2 times per month	N/A	21 (26.3)
	1–2 times per week		23 (28.7)
	3+ times per week		16 (20.0)

**Table 7:** Mean scores for consumer acceptance of raw and blanched kelp salad on a 9-point hedonic scale (mean ± SD (n = 102)).

Attributes	Raw	1 min Blanch	3 min Blanch
Appearance	6.3 ± 1.5 ^a^	6.5 ± 1.6 ^a^	6.6 ± 1.4 ^a^
Color	6.1 ± 1.7 ^b^	6.5 ± 1.4 ^a^	6.5 ± 1.4 ^ab^
Texture	6.4 ± 1.5 ^a^	6.5 ± 1.4 ^a^	6.6 ± 1.6 ^a^
Flavor	5.5 ± 1.9 ^b^	6.5 ± 1.7 ^a^	6.6 ± 1.7 ^a^
Overall liking	5.7 ± 1.7 ^b^	6.5 ± 1.7 ^a^	6.5 ± 1.7 ^a^

Each value is the mean ± standard deviation (n = 102). Superscripts: different small letters within rows indicate significant difference among treatments (*p* ≤ 0.05). 1 = Dislike Extremely and 9 = Like Extremely.

**Table 8:** Descriptors selected for each kelp salad treatment (n = 102).

Descriptors	Raw Kelp	1 min Blanched Kelp Salad	3 min Blanched Kelp Salad
Chewy	27	26	29
Firm	23	25	28
Tender	23	21	15
Juicy	7	15	10
Mushy	9	8	9
Soft	8	6	8
Tough	5	1	3

**Table 9:** Mean scores for consumer acceptance of blanched fresh-, blanched frozen- and cabbage sauerkraut on a 9-point hedonic scale.

Attributes	Sauerkraut		
	Raw Cabbage	Blanched Kelp	Blanched/Frozen Kelp
Appearance	6.7 ± 1.4 ^a^	6.5 ± 1.6 ^a^	6.3 ± 1.6 ^a^
Color	6.5 ± 1.5 ^a^	6.5 ± 1.5 ^a^	6.3 ± 1.5 ^a^
Aroma	6.3 ± 1.6 ^a^	5.5 ± 1.8 ^b^	5.7 ± 1.8 ^ab^
Flavor	6.8 ± 1.4 ^a^	5.9 ± 1.9 ^b^	6.1 ± 1.8 ^b^
Texture	7.0 ± 1.3 ^a^	6.8 ± 1.4 ^a^	6.7 ± 1.4 ^a^
Overall liking	6.8 ± 1.4 ^a^	6.0 ± 1.9 ^b^	6.1 ± 1.7 ^b^

Each value is the mean ± standard deviation (n = 80). Superscripts: different small letters within rows indicate significant difference among treatments (*p* ≤ 0.05). 1 = Dislike Extremely and 9 = Like Extremely.

**Table 10:** Descriptors selected for each sauerkraut treatment based on a Check-all-that-apply question (CATA) ^a.^

Descriptors	Cabbage Sauerkraut	Blanched Fresh Sauerkraut	Blanched/Frozen Sauerkraut
Crunchy	54	53	45
Pickled	54	46	42
Sour	42	38	31
Salty	37	56	50
Traditional kraut	35	5	9
Fresh	34	20	20
Tangy	31	30	27
Clean	17	13	9
Pungent	13	21	14
Boiled cabbage	12	8	8
Well rounded	10	6	14
Bland	7	0	0
Ocean breeze	6	24	23
Sweet	6	4	6
Mild	6	2	12
Bitter	5	11	10
Fizzy	4	3	2
Metallic	3	8	7
Mellow	3	2	4
Brackish	2	18	17
Fishy	2	24	22
Musty	2	2	4
Soggy	2	3	3
Slimy	2	8	9
Soft	1	5	5
Mushy	0	2	5

^a^ CATA = choose all that apply. Values shown are counts. Participants could check as many descriptors as they wished.

## Data Availability

The datasets generated during and/or analyzed during the current study are available from the corresponding author on reasonable request.
